# Mediators of Inflammation well on track to connect Molecular Pharmacology and Therapeutics

**DOI:** 10.1155/S0962935195000019

**Published:** 1995-01

**Authors:** Iván L. Bonta


Mediators of Inflammation 4, 3 (1995)

### Mediators of Inflammation well on track to connect Molecular Pharmacology and Therapeutics

*Mediators of Inflammation* has now completed its first three years of publication and this prompts me to address our journal's readers.

First of all, let me recount some of the very good things that have marked these three years. There has been an appreciable increase in the submission of unsolicited articles. It is also a good sign that, following the publication of their first paper, several authors have submitted a second paper to our journal. The constant flow of submissions has made the regular bimonthly appearance possible. This, in turn, has contributed to the fact that *Mediators of Inflammation* has qualified for a listing in *Current Contents* and being covered by major abstracting services, despite its short existence. Authors can thus be assured of a broad readership of their articles.

All of this would not have been possible without the permanent and creative assistance of members of the Editorial Board and external referees. I would like to take the opportunity of thanking those members who are retiring from the journal and to welcome those who, following our invitation, are joining the Editorial Board. The enlargement of the Editorial Board has become necessary to enable us to cope with the flexible profile of our journal.

I would like to elaborate a little on what we understand by the flexible profile of our journal. Ever since its launch, in contrast with several more specialized journals that focus on a particular class of inflammatory mediator (e.g. eicosanoids, PAF, histamine, cytokines, etc.), the inherent power of *Mediators of Inflammation* has been obvious. It is expected to continue flourishing even when some individual mediators take a back seat (as they inevitably do) and are replaced by newly emerging ones. Indeed, the scene changes very rapidly. The declining interest in prostanoids and the continuing progress of cytokines and adhesion molecules exemplify the fluctuations of the field. Our journal easily keeps pace with these and related trends.

It is also important to devote a few words to the subtitle of the journal. The present issue is the first to carry on its masthead the subtitle *Molecular Pharmacology and Therapeutics*. The introduction of this subtitle does not announce a new departure for our journal, but simply reflects its true nature. Many scientific domains are well represented in our journal, including experimental disciplines (pharmacology, immunology, cell biology, etc.) **as well as** contributions from clinical areas. Clearly, this indicates that mediators of the inflammation process are of pivotal importance in a great many fields of the life sciences, and that our journal is on its way to developing into a keynote forum for disseminating knowledge in all these domains.

The backbone of our journal remains the publication of full-length original *Research Articles*. However, as echoes from readers indicate the usefulness of *Invited Reviews*, we shall be continuing our efforts to publish this kind of paper. Also we wish to introduce two new categories of paper, namely *Short Communications* and *Letters to the Editors*. For the scope and style of these, we refer the reader to the revised *Instructions to Authors*.

One of the original goals set by *Mediators of Inflammation* was for our journal to become a prime forum of the highest quality in the field of research on inflammation. We are still some distance from this goal, mainly because the scientific standard of the research articles was occasionally variable. Now that the flow of submitted articles has increased appreciably, it is appropriate to raise the standards for acceptance, made necessary by the fierce competition between journals for really excellent papers.

Finally, I would like to emphasize two features, neither of which is unfamiliar to contributors and readers of our journal. One of these is speed in refereeing and publishing. In close collaboration with the publishers, Rapid Communications of Oxford, all possible efforts will be continued to serve the research community in disseminating the latest results with the highest speed. The other feature maintained by our journal is the **Janus principle** of the mediators' concept. This principle of duality, namely that mediators can exert both harmful and beneficial functions in health and disease, gathers momentum in our journal. When some of the articles advocating this principle appear to be controversial, they should provoke counteropinions amongst colleagues. Such *Debate Articles* are to be encouraged.

In the coming years, my colleague Editors and I look forward to receiving the best and latest results from the scientific community of the inflammation field.

*Iván L. Bonta*
*Editor-in-Chief*

© 1995 Rapid Communications of Oxford Ltd

